# The Influence of Cyclic Torsion with Application of Current Pulses on the Formability of CuZn30 Brass

**DOI:** 10.3390/ma18163912

**Published:** 2025-08-21

**Authors:** Zbigniew Zimniak, Wojciech Weiler, Karol Jaśkiewicz

**Affiliations:** Department of Metal Forming, Welding and Metrology, Wrocław University of Science and Technology, 7–9 Ignacego Łukasiewicza Street, 50-371 Wrocław, Polandkarol.jaskiewicz@pwr.edu.pl (K.J.)

**Keywords:** plastometric tests, cyclic torsion, current pulses, electrically assisted forming, CuZn30 brass

## Abstract

This article presents a study on symmetric cyclic torsion with the application of electric pulses and their effect on the formability of α-brass CuZn30 at room temperature. Preliminary tests were carried out using a conventional monotonic torsion test. The obtained results served as a reference for the subsequently conducted symmetric cyclic torsion tests. Then, under analogical deformation conditions, tests were conducted with the application of electric pulses featuring various parameters: different pulse durations and different periods, i.e., intervals between successive pulses. Microstructural studies of the deformed material were conducted, including examinations using a microscope equipped with an electron backscatter diffraction (EBSD) detector. Based on the results, it was found that the application of electric pulses during cyclic torsion tests consistently leads to a reduction in stress compared to cyclic torsion tests conducted at ambient temperature without current flow. In most cases, it also results in an increase in strain compared to tests without the application of electric pulses. The electroplastometric torsion tests carried out in this study within the bulk forming process are the first of their kind to combine cyclic torsion with electrically assisted forming (EAF). The proposed combination may lead to the development of new deformation methods in real manufacturing processes.

## 1. Introduction

The application of electric current—both direct and pulsed—during plastic deformation of metallic materials can significantly reduce stress, increase strain, and alter various other properties of metals. Electrically assisted forming (EAF) processes, which are part of the broader concept of electrically assisted manufacturing (EAM), are currently attracting significant interest, leading to new research in this field. In a number of experimental studies, attempts have been made to explain the nature of the phenomena occurring during the plastic deformation of metals assisted by electric current flow. However, the physical phenomena responsible for the increase in strain and the reduction in stress in electrically assisted processes have not yet been clearly explained [1,2,3].

The most common theories describing the changes in material behavior occurring during plastic forming processes assisted by electric current flow are divided into thermal and athermal theories. Thermal theories are related to the heat generated during current flow. Athermal theories are associated with other broadly understood phenomena linked to current flow and are referred to as the electro-plastic effect (EPE). This effect occurs during the simultaneous plastic deformation and flow of electric current [4].

The main athermal theories include the electron wind theory [2,5], the theory of magnetoplasticity [6,7,8], and the theory of excess free electrons [9,10]. The most common thermal theories include the electron stagnation theory [11,12], the theory of bulk Joule heating, and the theory of localized Joule heating.

Experimental studies involving the application of electric pulses are most commonly conducted using uniaxial tensile tests [13,14,15,16,17,18,19,20,21,22,23,24].

Shang et al. [13] investigated the coupled effects of electric pulse, temperature, strain rate, and strain magnitude on the plasticity of 5182-O aluminum alloy. Tensile tests with and without electropulsing were conducted at the same temperature. The results showed that under the influence of electric pulses, the elongation increased dramatically and the flow stress decreased.

In ref. [14], a study on the electro-plastic tension of aluminum alloy 5754-H111 was described. The authors demonstrated that the use of current pulses significantly improved the plasticity of the tested material, with its elongation nearly doubling compared to tensile tests without current flow. The increase in elongation was more pronounced at lower strain rates.

Farkhondeh et al. [15] analyzed the influence of electric current on the uniaxial tensile testing of AA6061 aluminum alloy sheets subjected to two different heat treatments: T6 and annealing. The authors demonstrated that the electroplastic effect increases both the material’s formability and its strength, which is more pronounced under annealed conditions. In the T6 state, the application of electric current resulted in a 5.7% increase in formability, a 3.11% increase in ultimate tensile strength, and a 3.79% increase in yield strength. In the annealed state, formability increased by 23.4% and yield strength by 20.71%, while ultimate tensile strength did not improve.

Shasha et al. [16] investigated the effects of electrical pulse coupling, temperature, strain rate, and strain on the flow properties and plasticity of 7075-T6 aluminum alloy. The authors showed that the yield strength decreases with increasing current density. Compared with traditional furnace heating, electrically assisted heating reduces the yield strength and increases elongation. The higher the current density, the greater the coefficient of electroplastic effect. With increasing current density, the springback angle decreases, the rebound damping effect improves, the bending stress decreases with increasing current density, and the stress-plastic cross section decreases significantly.

Korolkov et al. [18] investigated the deformation of single-phase titanium Grade 4 and two-phase VT6 alloy under tensile loading with external heating and pulsed current. The influence of the electroplastic effect on the reduction in yield stress under tensile loading at the same temperature was higher than that of the heating effect, for both Grade 4 and the VT6 alloy. This confirms the athermal nature of the pulsed current’s influence. The decrease in yield stress was smaller for the VT6 alloy than for Grade 4, with the same pulsed current modes.

Kinsey et al. [19] performed tensile tests on titanium alloy Ti6Al4V (Grade 5) and stainless steel 304SS at a very high strain rate of 1000 s^−1^ with simultaneous direct current flow at densities ranging from 60 to 180 A/mm^2^. The authors found that the application of current at such high strain rates has no effect on the material’s plasticity because the dislocation movement speed is already so high that the electric current cannot further increase it.

On the other hand, studies [20,21] show that in some metallic materials deformed with the application of electric pulses, changes occur that cannot be explained using the thermal and athermal theories presented above. Too low a current density disqualifies the athermal theories, while too low a temperature does not allow the observed changes to be justified by thermal theories.

Olevsky et al. [22] developed a unique method of electric nano pulsing (ENP) and built a device that generates electric pulses with very high current density (up to 10^12^ A/m^2^), ultrashort duration (<1 μs), and frequency up to 100 Hz. The ENP method allows delivering electric current to the tested material within nanoseconds, enabling an almost instantaneous increase in Joule heating and a very strong electric field effect.

Fan et al. [23] performed tensile tests on α-brass CuZn30 at a strain rate of 0.003 s^−1^ with the application of direct current at a density of 50 A/mm^2^. They observed that the current flow caused melting at the grain boundaries. The maximum temperature of the sample during the tests was about 60% of the melting temperature of CuZn30 brass—therefore, it was concluded that the cause of the melting was localized Joule heating concentrated at the grain boundaries. Similar phenomena occurring during electric pulse processing of brass were described by Wang et al. [12], as well as Majumdar et al. [24], who studied aluminum bronzes.

In recent years, research on the effects of electric current flow in sheet metal stamping processes has been initiated, where the main challenge is the high heat absorption by the tools. For this reason, these experiments are very difficult, which results in only a few researchers working on electrically assisted forming in stamping [25,26,27].

Article [25] described the influence of electric pulses on the formability and microstructure of aluminum 5754 alloys. The application of electric pulses caused an increase in punch penetration from 14% to over 26%.

Valoppi et al. [26] demonstrated that the flow of electric current significantly reduced the stamping force of Ti6Al4V (Grade 5) sheets, increased the material’s formability, improved the geometric accuracy of the product, and caused an increase in surface hardness and roughness.

Lv et al. [27] demonstrated that the application of electric current increased the height of the steel sheet drawpiece. Products formed with simultaneous electric current flow showed hardness in some areas up to 10% higher than those drawn without current flow.

Electropulsation is also used in sheet metal rolling and punching processes [28,29,30]. Wang et al. [28] studied the electroplastic rolling of magnesium alloys with rare earth elements: Gd, Y, and Zr. These alloys are characterized by poor formability, which is manifested by edge cracks during rolling. The authors compared hot rolling and electroplastic rolling and found that the application of electric pulses suppressed edge cracking in the Mg-13Gd-4Y-2Zn-0.5Zr alloy and improved the material’s formability, increased the nucleation rate, and accelerated dynamic recrystallization.

Zhang et al. [29] conducted research on the production of rolled composite plates from an Al and Mg alloy. Compared with hot rolling technology, the electrically assisted rolling process at room temperature showed a very significant improvement in the performance of interfacial bonding effects. A new structure with increased tensile strength was obtained.

Liu et al. [30] formed sheets in a punching process with the application of electric pulses. They studied a 1.5 mm thick sheet made of AZ31B magnesium alloy. Pulses with a current density ranging from 188 to 240 A/mm^2^ and a frequency from 200 to 400 Hz were applied. The application of pulsed current reduced the process force by up to 75% and increased the material’s plasticity.

Research on plastic forming processes with the application of electric current is conducted not only in the above-mentioned area of sheet forming but also in the field of bulk plastic deformation.

In articles [31,32], the authors carried out a wire drawing process with the application of electric pulses. In study [31], copper wire with a diameter of 1.85 mm was drawn through a die with a diameter of 1.68 mm at a speed of 0.7 m/s. The application of current with a density of 1000 A/mm^2^, pulse duration of 8 μs, and frequency of 7 Hz reduced the drawing force by 8% and increased elongation by over 30%, without lowering strength parameters.

Similar research was conducted by Tang et al. [32], who drew a wire made of 304L austenitic steel with a diameter of 1.6 mm at a speed of 6.5 m/min. During the tests, they applied pulsed current with a density of 1000 A/mm^2^, pulse duration of 20 μs, and frequencies ranging from 100 to 600 Hz. Under the influence of electric pulses, the drawing force was reduced by up to half, plasticity increased, and surface quality significantly improved, but at the same time, strength decreased.

Jones et al. [33] conducted forging with simultaneous electric current flow. Cylindrical preforms with a diameter of 7.94 mm and a height of 11.94 mm were made from AZ31B-O magnesium alloy. The application of current with a density of 30 A/mm^2^ and higher clearly increased the plasticity of the processed material and reduced the stress. Similar studies, but for steel and titanium, aluminum, and copper alloys, were conducted by Perkins et al. [34]. Steel and copper alloys showed only a slight increase in plasticity, while aluminum and titanium alloys produced better results, comparable to those obtained in study [33].

A very interesting example of electroplastic processing is compression and microforming. Korolkov et al. [35] studied titanium Grade 4 in a compression process assisted by electric current flow. The current flow caused a 27% reduction in flow stress, a 50% reduction in the conditional yield strength, a reduction in strain intensity, an increase in microhardness, and partial dissolution of intermetallic particles, without affecting the grain size.

Liu et al. [36] proposed a new technology for electrically assisted microforming. Ultrathin-walled Inconel 718 capillaries are essential components of the heat transfer system in hypersonic, pre-cooled aircraft engines. The effectiveness of the designed process solution was initially verified through process experiments.

To summarize, the innovative technology of using electric current in metal forming processes alters their microstructure and mechanical properties. The application of current has various consequences for the plastic behavior of different metals. Current-induced phenomena primarily include improved deformability, reduced stress, and accelerated evolution of the material’s microstructure. The review presented above discussed sheet metal and bulk forming processes. However, it is worth noting that among the many studies on EAM processes [37,38], there is a lack of torsion tests.

In this study, the authors investigated, for the first time, CuZn30 brass in a symmetrical cyclic torsion test with the application of electric pulses. The electroplastic torsion tests were carried out on a torsion plastometer adapted to perform experiments with the application of current pulses.

## 2. Experimental Procedure

### 2.1. Tested Material

The CuZn30 brass intended for the study was cast in the form of ingots from copper and zinc. The ingots were extruded into round rods with a diameter of 20 mm. The chemical composition of the brass was determined using a GDS500A Glow Discharge Spectrometer, Leco, St. Joseph, MI, USA and is presented in Table 1.

**Figure 1:**
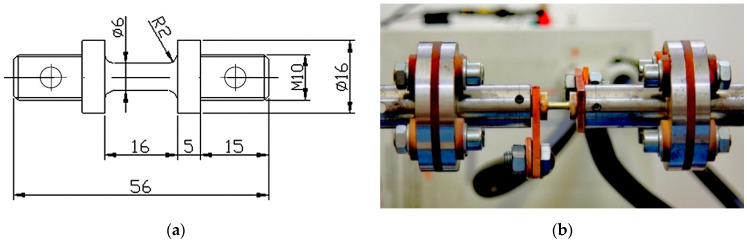
Sample dimensions (**a**) and the method of mounting the sample in clamps allowing for the application of electric current (**b**).

Because the material exhibited significant microstructural heterogeneity, the rods underwent homogenization annealing at 700 °C for 60 min. From the prepared material, cylindrical samples with flanges were machined to ensure an increased surface area for electric current flow in the gripping sections. The shape and dimensions of the sample are shown in Figure 1a.

The surface quality of the machined material and the condition of its surface layer significantly influence the strength properties of the manufactured components—in this case, the plastometric test samples. In materials strengthened by strain hardening—such as the α-brass CuZn30—deformation of the surface layer leads to changes in residual stress values. During turning, tensile stresses develop in the surface layer of the machined material ahead of the cutting tool edge, while compressive stresses appear behind the turning tool path. Therefore, before starting the tests, the turned samples were subjected to stress-relief annealing for 10 min at 700 °C to eliminate the stresses formed during turning. Then, the gauge section of each sample was ground with abrasive paper to remove the oxidized surface layer. The prepared samples were then deformed in torsion tests conducted on a custom-built torsion plastometer.

Metallographic samples were prepared in a plane parallel to the axis of the specimens. The CuZn30 brass was etched with a 10% water solution of ammonium persulfate (APS) for 2 min. The microstructure of the examined samples was observed using an optical microscope GX51, Olympus, Tokyo, Japan, and a scanning electron microscope VEGA3, TESCAN, Libušina, Czech Republic, equipped with a backscattered electron diffraction (EBSD) detector.

### 2.2. Research Equipment

The authors-designed torsion plastometer used in the study is equipped with special grips for holding the specimen, which are fitted with insulators (Figure 1b). The separation of the grips from the rest of the plastometer components by insulators allows for the safe delivery of electric current to the specimen and prevents the current from flowing to any other parts of the system besides the tested specimen. Electric pulses are supplied to the specimen via copper wires.

**Figure 2:**
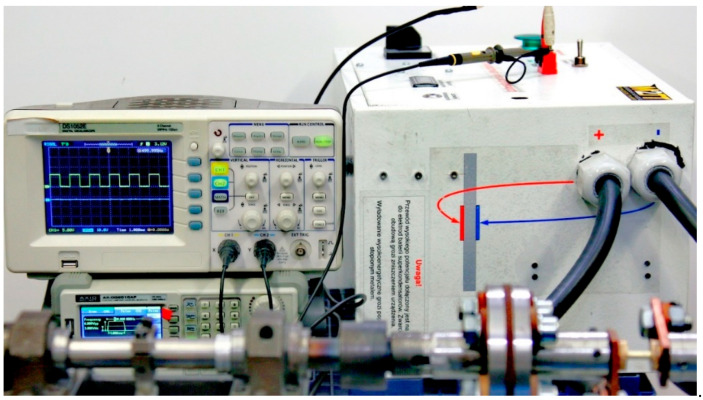
Oscilloscope, below it the function generator, and on the right side the high-current power supply.

A high-current power supply with a supercapacitor bank (Figure 2) was used to generate the electric current. The setup was equipped with an AX-DG2010AF, AXIOMET, Łódź, Poland, function generator to shape the pulses, a DS1052E, Rigol, Suzhou, China, digital oscilloscope to monitor the current pulses, and an RCT, Power Electronic Measurements, Nottingham, UK, coil to measure the electric current flowing through the specimen. The authors-designed current pulse generator design utilized supercapacitors with a total capacitance of 14,000 F and a maximum operating voltage of 2.7 V. In the experiments, the initial voltage before each test was 2.52 V.

### 2.3. Methodology

**Figure 3:**
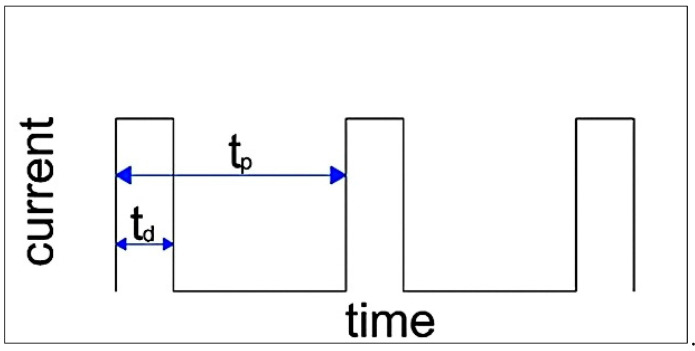
Shape and parameters of the electric current pulses.

Based on extensive preliminary tests conducted at room temperature with various strain rates and amplitudes, the following deformation variants of α-brass CuZn30 were selected for this study:

- conventional monotonic torsion with a strain rate of $\overset{\cdot }{\varepsilon }$ = 1 s^−1^,
- symmetric cyclic torsion with a strain rate of $\overset{\cdot }{\varepsilon }$ = 1 s^−1^ and a strain amplitude of A = 0.06,
- symmetric cyclic torsion with a strain rate of $\overset{\cdot }{\varepsilon }$ = 1 s^−1^ and a strain amplitude of A = 0.06, combined with the application of rectangular electric pulses with pulse duration t_d_ and period t_p_ (Figure 3).

In cyclic torsion with the application of electric pulses, the following current parameters were used: pulse durations t_d_ = 50 and 100 µs and periods t_p_ = 50, 75, 100, 150, 200, and 300 µs. The measured current density ranged from 127.4 to 135.4 A/mm^2^.

The reference results for those obtained in cyclic torsion without the application of current are the results from the conventional monotonic torsion test, while for cyclic torsion tests with the application of electric pulses, the reference results are those from cyclic tests without current flow, conducted at the same strain rate and amplitude.

In the torsion test, the torsional moment is measured as a function of the sample’s angle of twist, and these values are then converted into stress and strain. The precise determination of the stresss–train relationship depends on the used measurement data processing method as well as the accuracy of the deformation control system. In this study, the method applied to determine the relationship between the angle of twist and the stress and strain describes the flow stress with the following equation:

(1) $$\sigma_{p}=\sqrt{3}\tau =\sqrt{3}\frac{3M(m+n)}{2\pi r_{rz}^{3}}$$

The equivalent strain is described by the equation:

(2) $$\varepsilon_{red}=\frac{1}{\sqrt{3}}\gamma =\sqrt{3}\frac{r_{rz}\omega }{l_{rz}}$$

where:

*τ*—shear stress,
*M*—torsional moment,
*n*—strain hardening factor,
*m*—strain rate sensitivity factor,
*ω*—specimen torsional angle,
*r_rz_*—specimen true radius,
*l_rz_*—specimen true length,
*γ*—non-dilatational strain.

The strain hardening factor *n* is defined by the following equation:

(3) $$n=\frac{N}{M}\frac{\delta M}{\delta N}$$

The strain rate sensitivity factor *m* is defined by the following equation:

(4) $$m=\frac{\dot{N}}{M}\frac{\delta M}{\delta \dot{N}}$$

where:

*M*—torsional moment,
*N*—number of specimen rotations,
*Ṅ*—specimen torsion rate.

**Figure 4:**
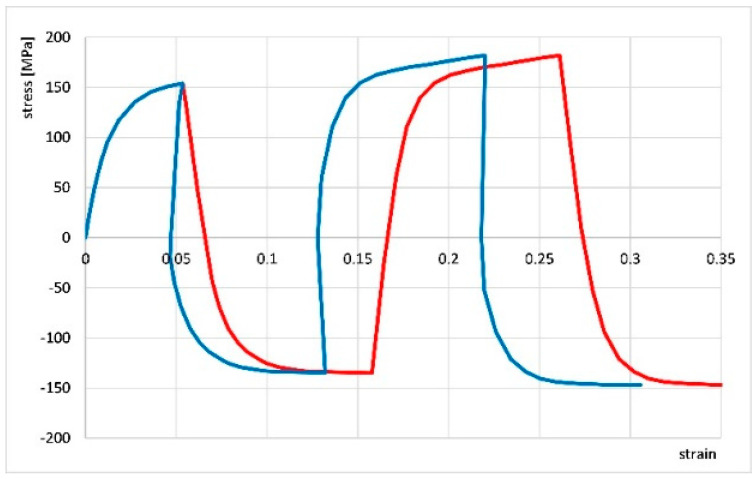
Comparison of the cyclic oscillatory torsion curve with strain amplitude A = 0.06 containing elastic strains (red line) with the true curve (corrected—blue line).

Most authors of existing studies on material behavior during cyclic torsion have not accounted for the division of strain into elastic and plastic components. However, the knowledge of elastic and plastic strains in successive strain hysteresis loops is essential for determining true stress–strain curves. In bulk forming processes, it is often assumed that elastic strains are so small compared to plastic strains that they can be neglected. However, during cyclic oscillatory torsion tests, elastic strain occurs in every deformation cycle and makes it difficult to read the plastic strain. Figure 4 compares stress curves for cyclic oscillatory torsion with a strain amplitude of 0.06 as a function of total strain (plastic strain + elastic strain, red line) and as a function of plastic strain only (blue line). The comparison shows that even for a small total strain—for example, 0.27—the share of elastic strains in a given test reaches about 20%, which leads to significant errors in the interpretation of the obtained results. With further deformation, this share increases. Therefore, a method for separating elastic and plastic strains was developed. A special computer program was prepared to calculate plastic strains in individual hysteresis loops of cyclic strains using this strain separation.

## 3. Results and Discussion

### 3.1. Results Without Application of Electropulsing

**Figure 5:**
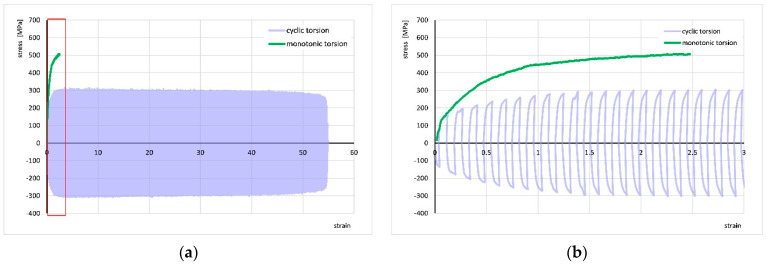
Comparison of stress–strain curves for monotonic torsion at a strain rate of $\overset{\cdot }{\varepsilon }$ = 1 s^−1^ and cyclic torsion at a strain rate of $\overset{\cdot }{\varepsilon }$ = 1 s^−1^ with a strain amplitude of A = 0.06 (**a**), and a close-up of the initial segment of the curves marked by the red box (**b**).

Figure 5 shows the stress–strain curve for monotonic torsion at a strain rate of $\overset{\cdot }{\varepsilon }$ = 1 s^−1^ and the stress–strain curve for cyclic torsion at the same strain rate and strain amplitude A = 0.06. The increase in strain compared to the monotonic test results is approximately 25-fold—the strain reached a value of about 55. Meanwhile, the stress decreased from approximately 500 MPa to about 300 MPa.

### 3.2. Results with Application of Electropulsing

**Figure 6:**
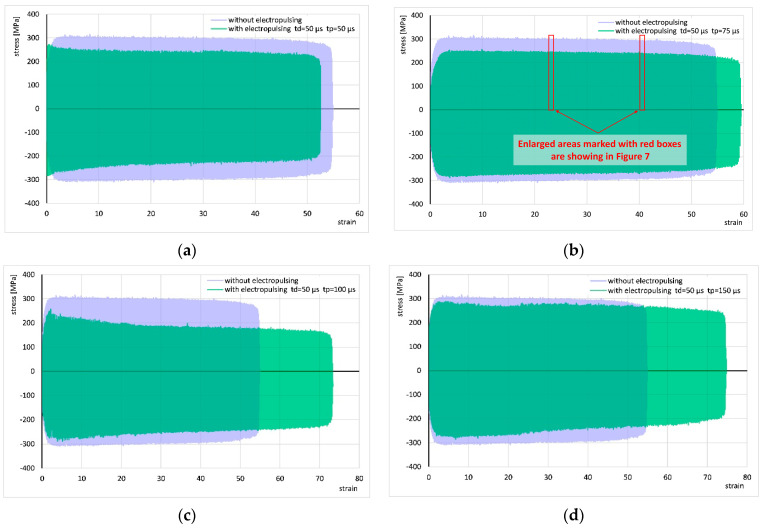
Comparison of the stress–strain curve of cyclic torsion without current flow and the stress–strain curves with the application of electric pulses with a duration of t_d_ = 50 µs and periods t_p_ = 50 µs (**a**), 75 µs (**b**), 100 µs (**c**), and 150 µs (**d**). Enlarged areas marked with red boxes are showing in Figure 7.

The results of cyclic torsion with the application of electric pulses with a duration of t_d_ = 50 µs and periods t_p_ = 50, 75, 100, and 150 µs are shown in Figure 6 and compared to corresponding results without current application. In all experiments, the stress decreased to about 20 MPa compared to the test without current. The flow of pulses with periods t_p_ = 100 and 150 µs led to a significant increase in strain, the test with a period of t_p_ = 75 µs showed a relatively small increase in strain, while with a period of t_p_ = 50 µs the strain decreased.

**Figure 7:**
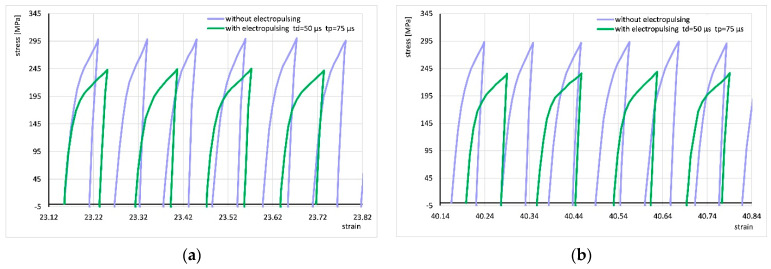
Comparison of fragments of stress–strain curves for cyclic torsion without current flow and with the application of electric pulses of duration t_d_ = 50 µs and period t_p_ = 75 µs at strains of approximately 23 (**a**) and approximately 40 (**b**). These are the areas marked in Figure 6b.

For better visualization of the strain cycle progressions, Figure 7 shows enlarged fragments of the stress–strain curve from Figure 6b (areas marked with red boxes). It can be seen that the application of the electric pulse changes the shape of the curve, which may suggest a change in the strain hardening factor. However, the shape of the curve in subsequent cycles does not change significantly with increasing strain. For all other applied electrical parameters, the shape of the cycle progression was very similar.

**Figure 8:**
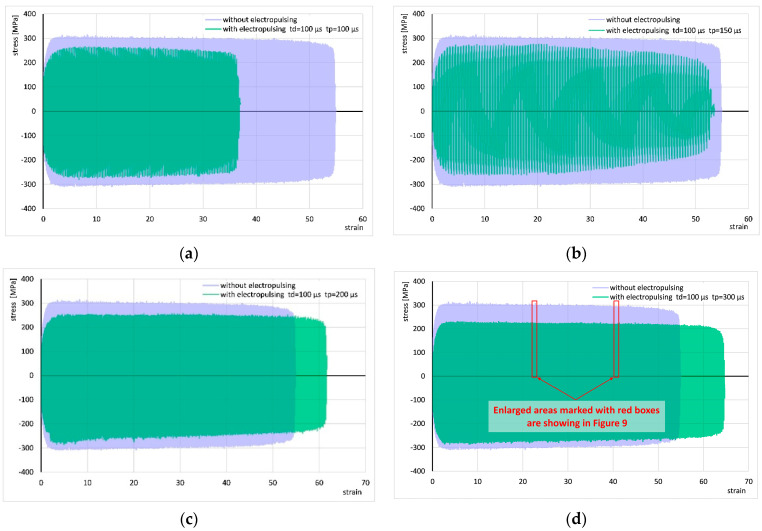
Comparison of the stress–strain curve of cyclic torsion without current flow and stress–strain curves with the application of electric pulses of duration t_d_ = 100 µs and periods t_p_ = 100 µs (**a**), 150 µs (**b**), 200 µs (**c**), and 300 µs (**d**). Enlarged areas marked with red boxes are showing in Figure 9.

The results of cyclic torsion with the application of electric pulses of duration t_d_ = 100 µs and periods t_p_ = 100, 150, 200, and 300 µs are presented in Figure 8 and compared to the corresponding results without current application. In these experiments, the stress decreased by approximately 25 to 30 MPa compared to the test without current application. The flow of pulses with periods t_p_ = 200 and 300 µs led to an increase in strain compared to tests without current flow, while the application of pulses with periods t_p_ = 100 and 150 µs caused a decrease in strain.

**Figure 9:**
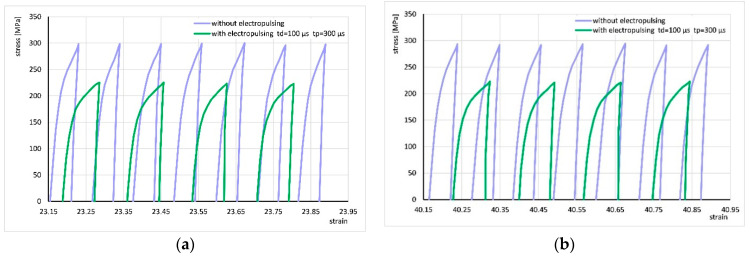
Comparison of fragments of stress–strain curves for cyclic torsion without current flow and with application of electric pulses of duration t_d_ = 100 µs and period t_p_ = 300 µs at strain of approx. 23 (**a**) and approx. 40 (**b**). These are the areas marked in Figure 8d.

Figure 9 shows the areas marked with red frames in Figure 8d. Similarly to the description above, the application of current changed the shape of the curve, which did not change significantly in subsequent cycles with increasing strain. For other applied electrical parameters, the shape of the cycle progression was similar.

**Figure 10:**
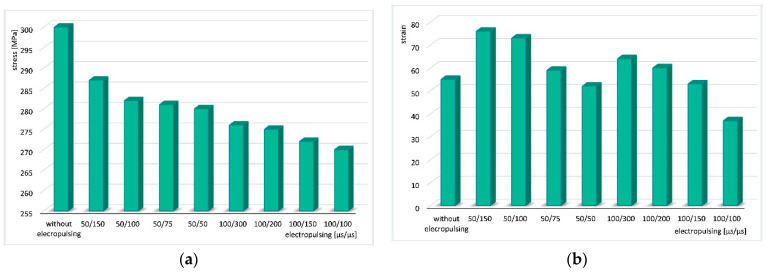
Change in stress (**a**) and strain (**b**) depending on the electric pulse parameters.

For better visualization of the obtained results, summary graphs were prepared showing changes in stress and strain depending on the parameters of the electric pulses (Figure 10). In Figure 10a, it is clearly visible that stress decreases with increasing pulse duration and shortening of its period. In cyclic torsion tests with current application, stress is always lower than in the analogical test without electric pulse application. The next graph (Figure 10b) shows changes in strain depending on the electric pulse parameters. The longer the period, the greater the strain value.

### 3.3. Microstructural Studies

**Figure 11:**
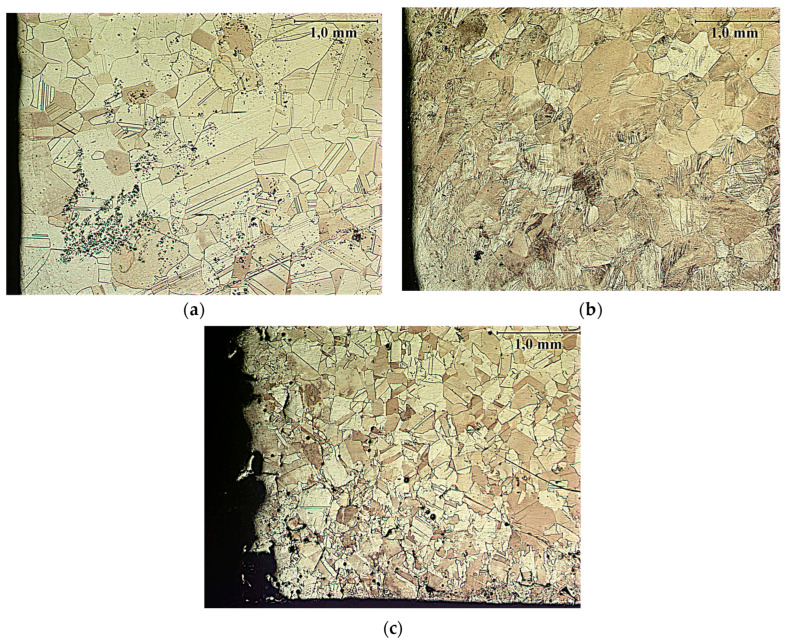
Microstructure of undeformed CuZn30 material (**a**), after cyclic torsion with strain rate $\overset{\cdot }{\varepsilon }$ = 1 s^−1^, strain amplitude A = 0.06, without electropulsing (**b**) and after cyclic torsion with strain rate $\overset{\cdot }{\varepsilon }$ = 1 s^−1^, strain amplitude A = 0.06, with electropulsing of duration t_d_ = 100 µs and period t_p_ = 100 µs (**c**).

The microstructure of the undeformed CuZn30 brass prepared for testing is shown in Figure 11a. This is a typical microstructure for α-brasses. It is characterized by the morphology of equiaxed grains of the α phase solution, featuring straight grain boundaries and the presence of twin boundaries. It is not a fine-grained microstructure—the grain size is varied. No presence of slip bands or grains with deformed shapes was observed.

The microstructure of the sample deformed by cyclic torsion without current application is shown in Figure 11b. This microstructure is characteristic of very large plastic deformations. The grains are roughly equiaxed, without twin boundaries that were previously visible in the undeformed material. Inside the grains, a clear accumulation of slip bands can be observed.

All microstructures of samples deformed by cyclic torsion with simultaneous electric pulse flow looked very similar, regardless of the used pulse parameters. An example of the microstructure image is shown in Figure 11c. At the sample fracture—where the greatest deformation occurred—the characteristic microstructure after very large plastic deformation is most visible. There is a noticeable significant grain fragmentation and a comparably much smaller presence of slip bands than after symmetric cyclic torsion without current. Grain boundaries are only slightly deformed. Grains remain relatively equiaxed and twin boundaries appear—this may indicate at least partial recrystallization of the material.

The average grain size was measured before and after deformation. The analysis was performed in accordance with ASTM E112-13 [39] and the appropriate grain size numbering G was given. The average grain size in undeformed brass was approx. 510 µm (G00). After cyclic torsion at a strain rate of 1 s^−1^ and amplitude of 0.06 without electropulsing, the average grain size was approx. 353 µm (G0). After cyclic torsion at the same strain rate and amplitude and electropulsing of duration t_d_ = 100 µs and period t_p_ = 100 µs, the average grain size was approx. 250 µm (G1).

### 3.4. Microstructure Analysis by EBSD

The sample deformed with the application of electric pulses of duration t_d_ = 100 µs and period t_p_ = 100 µs was selected for microstructure observation using the EBSD method, as these parameters of cyclic electroplastic deformation resulted in the lowest stress value.

**Figure 12:**
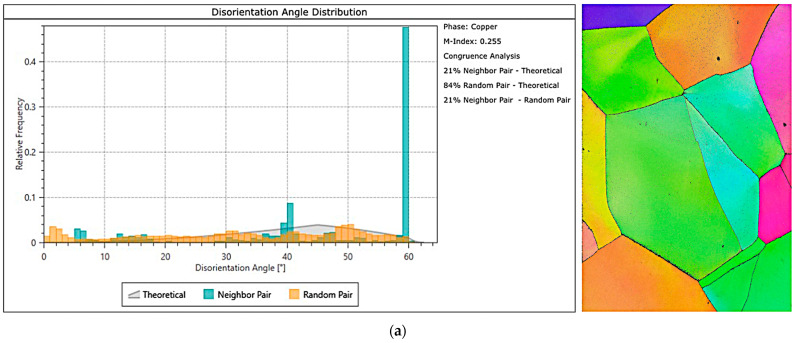

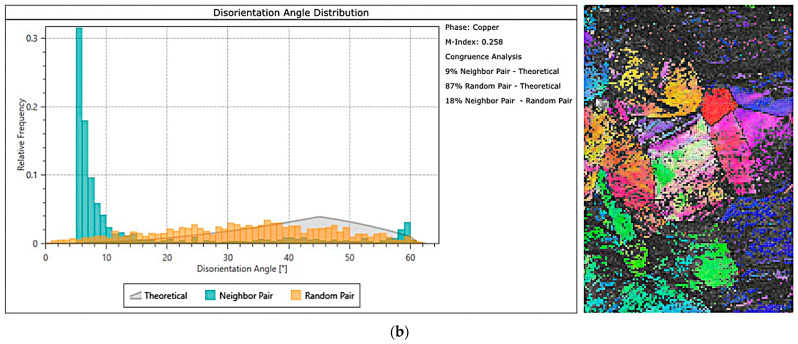
Comparison of the distributions of grains with low-angle and high-angle boundaries and EBSD maps IPF of the material: (**a**) undeformed and (**b**) cyclically deformed with the application of electric pulses.

Figure 12 presents the results of EBSD studies: disorientation angle distribution and inverse pole figure (IPF) map of the undeformed and electroplastically deformed material.

After torsion with electropulsing, grains were subdivided, and subgrains with low-angle boundaries, typical for subgrains, appeared. The distribution and fraction of grain boundaries changed. In the undeformed material, high-angle grain boundaries (around 60°) dominated. After deformation, low-angle boundaries (below 10°) are most prevalent. Originally large and equiaxed grains became deformed in regions where the greatest strains occurred.

The IPF map indicates the crystallographic orientation of individual grains relative to the observed surface. The grain orientation is visualized using colors: areas with the same orientation have the same color, selected from the base triangle of the stereographic projection, which is a fragment of the generated map. In the absence of deformation, grain orientations are random (Figure 12a), with no dominant directions.

Figure 12b shows the IPF map after cyclic torsion test with electropulsing, including the area near the edge of the broken sample, the location of the greatest deformation. Microstructure fragmentation and change in grain orientation are visible. In the dark area on the right side of the image, the crystalline microstructure of the material is so degraded that diffraction images of sufficient quality, i.e., good signal matching, cannot be obtained. This is not due to errors during testing, but to the severely damaged microstructure of CuZn30 after torsion, i.e., after very significant plastic deformation. The EBSD detector does not recognize such a severely damaged microstructure, hence the lack of signal matching, manifested in the black areas. This illustration nevertheless has cognitive value, especially as the available literature does not present EBSD maps of the material after torsion.

Microhardness measurements were also conducted on samples deformed with the application of electric pulses and compared with the microhardness measured on a sample deformed under the same conditions but without the electric current. The measurements were taken in the same areas and at a constant distance from the surface layer of the tested samples. The use of electroplastic torsion caused an increase in the microhardness of the material. The microhardness of the sample cyclically deformed without electric pulses was 169 HV, while in the torsion tests with the application of electric pulses it ranged from 198 to 228 HV.

## 4. Conclusions

This study investigated the influence of cyclic deformation and the application of electric pulses on the stress and strain of α-brass CuZn30. A method was used to present the results that allows the elimination of elastic strains from total strains, thereby obtaining the true stress–strain curves.

The application of electric pulses during cyclic torsion tests caused a reduction in stress compared to tests without current flow. For the applied electrical parameters, the stress decreased in the range of 4 to 10%, depending on the pulse duration and period. In most tests, the application of electric pulses resulted in an increase in strain compared to tests without current—the maximum increase in strain reached 38%. However, for three sets of pulse parameters, the strain decreased—by up to 33%.

The results of cyclic torsion tests with the application of electric pulses showed that the stress decreases with increasing pulse duration t_d_ and shortening of the pulse period t_p_. Changes in strain during tests with electric pulse application were irregular—the current flow during sample deformation could either significantly improve the plasticity of CuZn30 brass compared to tests without current, or worsen it. Nevertheless, the results indicate a clear trend: strain increases with shorter pulse durations t_d_ and longer pulse periods t_p_.

Metallographic studies showed that cyclic deformation without the application of electric pulses leads to the localization of strains in shear bands. In contrast, cyclic deformation with the application of electric pulses caused significant microstructural changes in the material and affected the proportion of grains with low-angle and high-angle boundaries.

The conducted studies showed that symmetric cyclic deformation, both without the application of electric pulses and with simultaneous current flow, significantly increases the strain and reduces the stress compared to monotonic deformation. The selection of appropriate electrical parameters allows control over the strain and stress levels of CuZn30 brass.

The results suggest that the application of suitable electroplastic deformation methods in industrial processes can lead to improved plasticity of this alloy. It is thus possible to choose process parameters that result in a significant decrease in stress, which is important for designing energy-efficient plastic forming processes requiring lower deformation forces. At the same time, there is an opportunity to select parameters that ensure very high strain values, opening perspectives for using these methods in processes where large material deformations are needed.

The electroplastic effect refers to an intrinsic mechanism by which an electric current significantly changes the mechanical properties of metals. According to the authors, the previously mentioned electron wind theory is one of the fundamental principles underlying this effect; however, the interaction of multiple concurrent phenomena makes it difficult to provide a precise and unambiguous explanation of electron–dislocation interactions. This theory assumes an interaction between electrons and dislocations in a metal through which a current is flowing. While Joule heating during the current’s passage through the material affects its plasticity, research has shown that the electroplastic effect also occurs at current parameters that do not cause a significant temperature increase.

According to this theory, the plastic deformation of a material sets its dislocations in motion. When the velocity of electrons flowing through the metal is greater than the velocity of the moving dislocations, and the vectors of both velocities are aligned, the electrons exert a specific force on the dislocations, accelerating them and facilitating their movement. This phenomenon is referred to as electron wind.

The authors state that it is necessary to intensify research into the mechanism of electroplasticity and establish an unambiguous theory that would provide guidance for the subsequent improvement of the electroplastic processing method.

The results suggest that the application of suitable electroplastic deformation methods in industrial processes can lead to improved plasticity of this alloy. It is thus possible to choose process parameters that result in a significant decrease in stress, which is important for designing energy-efficient plastic forming processes requiring lower deformation forces. At the same time, there is an opportunity to select parameters that ensure very high strain values, opening possibilities for using these methods in processes where large material deformations are needed.

## Figures and Tables

**Table 1:** Percentage chemical composition of the tested CuZn30 brass.

Material	Cu	Zn	Fe	Ni	As	Mn
CuZn30	70.1	residue	0.01	0.001	0.001	0.004
	Bi	Sn	Al	Si	Cd	Sb
	0.001	0.03	0.001	0.001	0.001	0.001

## Data Availability

The original contributions presented in this study are included in the article. Further inquiries can be directed to the corresponding author.

## References

[1] Dimitrov N.K., Liu Y., Horstemeyer M.F. (2020). Electroplasticity: A review of mechanisms in electro-mechanical coupling of ductile metals. Mech. Adv. Mater. Struct..

[2] Nguyen-Tran H.D., Oh H.S., Hong S.T., Han H.N., Cao J., Ahn S.H., Chun D.M. (2015). A review of electrically-assisted manufacturing. Int. J. Precis. Eng. Manuf.-Green Technol..

[3] Roth J.T., Loker I., Mauck D., Warner M., Golovashchenko S.F., Krause A. (2008). Enhanced formability of 5754 aluminum sheet metal using electric pulsing. Transactions of the North American Manufacturing Research Institution of SME.

[4] Ruszkiewicz B.J., Grimm T., Ragai I., Mears L., Roth J.T. (2017). A Review of Electrically-Assisted Manufacturing with Emphasis on Modeling and Understanding of the Electroplastic Effect. J. Manuf. Sci. Eng..

[5] Kravchenko V.Y. (1967). Effect of Directed Electron Beam on Moving Dislocations. J. Exp. Theor. Phys..

[6] Golovin Y.I. (2004). Magnetoplastic effects in solids. Physic Solid State.

[7] Molotskii M.I., Fleurov V. (1995). Magnetic effects in electroplasticity of metals. Phys. Rev..

[8] Molotskii M.I. (2000). Theoretical basis for electro- and magnetoplasticity. Mater. Sci. Eng..

[9] Li D., Yu E. (2011). An approach based on the classical free-electron theory to study electroplastic effect. Adv. Mater. Res..

[10] Li D., Yu E., Liu Z. (2012). Mechanism research and progress of metal’s pure electroplastic effect. Appl. Mech. Mater..

[11] Ruszkiewicz B.J., Mears L., Roth J.T. (2018). Investigation of Heterogeneous Joule Heating as the Explanation for the Transient Electroplastic Stress Drop in Pulsed Tension of 7075-T6 Aluminum. J. Manuf. Sci. Eng..

[12] Wang X.L., Guo J.D., Wang Y.M., Wu X.Y., Wang B.Q. (2006). Segregation of lead in Cu-Zn alloy under electric current pulses. Appl. Phys. Lett..

[13] Shang H., Wang S., Lou Y. (2024). Modeling and characterization on electroplastic effect during dynamic deformation of 5182-O aluminium alloy. Trans. Nonferrous Met. Soc. China.

[14] Dobras D., Zimniak Z., Zwierzchowski M., Dziubek M.A. (2024). Effect of strain rate on the mechanical behavior of Al-Mg alloy under a pulsed electric current. Metall. Mater. Trans. A Phys. Metall. Mater. Sci..

[15] Farkhondeh A., Bakhshi-Jooybari M., Gorji H., Mirnia M. (2025). Electroplastic Effect on Plastic Behavior of AA6061 Sheet in T6 and Annealed States. Int. J. Eng..

[16] Shasha D., Zhuang L., Zhijun L., Haojie S., Kang Z., Jiansheng X. (2025). Mechanical Properties of 7075-T6 Aluminum Alloy in Electrically Assisted Forming. Metals.

[17] Zimniak Z., Dobras D. (2019). Electroplastic effect of high manganese austenitic steel. Arch. Metall. Mater..

[18] Korolkov O.E., Pakhomov M.A., Stolyarov V.V. (2023). Electroplastic Effect in Titanium Alloys Under Tension. Inorg. Mater. Appl. Res..

[19] Kinsey B., Cullen G., Jordan A., Mates S. (2013). Investigation of electroplastic effect at high deformation rates for 304SS and Ti-6Al-4V. CIRP Ann. Manuf. Technol..

[20] Zhao S., Zhang R., Chong Y., Li X., Abu-Odeh A., Rothchild E., Chrzan D.C., Asta M., Morris J.W., Minor A.M. (2021). Defect reconfiguration in a Ti–Al alloy via electroplasticity. Nat. Mater..

[21] Zhu Y.H., To S., Lee W.B., Liu X.M., Jiang Y.B., Tang G.Y. (2009). Effects of dynamic electropulsing on microstructure and elongation of a Zn-Al alloy. Mater. Sci. Eng..

[22] Olevsky E.A., Jiang R., Xu W., Maximenko A., Grippi T., Torresani E. (2024). Quasi-instantaneous materials processing technology via high-intensity electrical nano pulsing. Nat. Sci. Rep..

[23] Fan R., Magargee J., Hu P., Cao J. (2013). Influence of grain size and grain boundaries on the thermal and mechanical behavior of 70/30 brass under electrically-assisted deformation. Mater. Sci. Eng..

[24] Majumdar A., Carrejo J.P., Lai J. (1993). Thermal imaging using the atomic force microscope. Appl. Phys. Lett..

[25] Dobras D., Zimniak Z., Zwierzchowski M., Dziubek M.A. (2023). Electrically-assisted deep drawing of 5754 aluminium alloy sheet. Proceedings of the 26th International ESAFORM Conference on Material Forming.

[26] Valoppi B., Sánchez Egea A.J., Zhang Z., González Rojas H.A., Ghiotti A., Bruschi S., Cao J. (2016). A hybrid mixed double-sided incremental forming method for forming Ti6Al4V alloy. CIRP Ann. Manuf. Technol..

[27] Lv Z., Zhou Y., Zhan L., Zang Z., Zhou B., Qin S. (2021). Electrically assisted deep drawing on high-strength steel sheet. Int. J. Adv. Manuf. Technol..

[28] Wang D., Qin S., Guo C., Chen H., Xiao L., Ren W., Sun J., Wang P., Hao L., Huang H. (2025). Deformability enhancement of rare earth magnesium alloy during electroplastic rolling. Mater. Sci. Eng. A.

[29] Zhang T., An X., Wang Y., Bian G., Wang T. (2025). Coordinated Regulation of Bonding Interfacial Structure and Mechanical Properties of Al/Mg Alloy Composite Plates by Electrically Assisted Rolling. Chin. J. Mech. Eng..

[30] Liu K., Dong X., Shi W., Wang X., Wu G. (2019). Investigation on Two Electrically-Assisted Forming Processes of AZ31B Magnesium Alloy Sheets. J. Shanghai Jiaotong Univ..

[31] Zimniak Z., Radkiewicz G. (2008). The electroplastic effect in the cold-drawing of copper wires for the automotive industry. Arch. Civ. Mech. Eng..

[32] Tang G., Zhang J., Zheng M., Zhang J., Fang W., Li Q. (2000). Experimental study of electroplastic effect on stainless steel wire 304L. Mater. Sci. Eng..

[33] Jones J.J., Mears L., Roth J.T. (2012). Electrically-assisted forming of magnesium AZ31: Effect of current magnitude and deformation rate on forgeability. J. Manuf. Sci. Eng..

[34] Perkins T.A., Kronenberger T.J., Roth J.T. (2007). Metallic forging using electrical flow as an alternative to warm/hot working. J. Manuf. Sci. Eng..

[35] Korolkov O.E., Misochenko A.A., Stolyarov V.V. (2024). Electroplastic Effect in Titanium Under Compression. Inorg. Mater. Appl. Res..

[36] Liu Y., Meng B., Zhao R., Wan M., Chen L. (2025). Design and Validation of a Novel Process Solution for Electrically Assisted Superalloy Capillary Microforming. Int. J. Precis. Eng. Manuf.-Green Technol..

[37] Lv Y., Chen G., ·Zhang B., Li H.J. (2024). Application of electroplastic effect in mechanical processing. Int. J. Adv. Manuf. Technol..

[38] Kim M.-J., Bui-Thi T.-A., Kang S.-G., Hong S.-T., Han H.N. (2024). Electric current-induced phenomena in metallic materials. Curr. Opin. Solid State Mater. Sci..

[39] (2021). Standard Test Methods for Determining Average Grain Size.

